# Bis(μ-*N*,*N*′-di-3-pyridylpyridine-2,6-dicarboxamide)bis­[dichloridomercury(II)] *N*,*N*-dimethyl­formamide disolvate

**DOI:** 10.1107/S1600536808040269

**Published:** 2008-12-06

**Authors:** Li-Hua Huang, Jie Wu

**Affiliations:** a Department of Chemistry, Zhengzhou University, Zhengzhou 450052, People’s Republic of China

## Abstract

The asymmetric unit of the binuclear title complex, [Hg_2_Cl_4_(C_17_H_13_N_5_O_2_)_2_]·2C_3_H_7_NO, contains one-half of the centrosymmetric mol­ecule and one dimethyl­formamide solvent mol­ecule. The Hg^II^ atom is four-coordinated by two N atoms from two ligands and two Cl atoms in a distorted tetra­hedral coordination geometry. Intra­molecular N—H⋯O hydrogen bonds may be effective in the stabilization of the structure. In the crystal structure, π–π contacts between pyridine rings [centroid-to-centroid distances 3.629 (3) and 3.595 (3) Å] may further stabilize the structure.

## Related literature

For general background, see: Ockwig *et al.* (2005 ▶); Qin *et al.* (2003 ▶); Baer *et al.* (2002 ▶). For bond-length data, see: Allen *et al.* (1987 ▶).
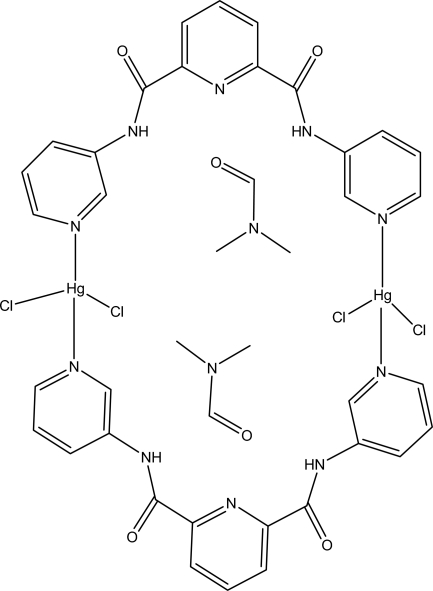


## Experimental

### 

#### Crystal data

[Hg_2_Cl_4_(C_17_H_13_N_5_O_2_)_2_]·2C_3_H_7_NO
*M* *_r_* = 1327.82
Triclinic, 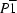

*a* = 7.4947 (15) Å
*b* = 12.262 (3) Å
*c* = 13.284 (3) Å
α = 79.79 (3)°
β = 73.74 (3)°
γ = 76.21 (3)°
*V* = 1130.2 (5) Å^3^
*Z* = 1
Mo *K*α radiation
μ = 7.08 mm^−1^
*T* = 294 (2) K
0.20 × 0.18 × 0.17 mm

#### Data collection

Rigaku Saturn 724 diffractometer
Absorption correction: numerical (*CrystalClear*; Rigaku/MSC, 2006 ▶) *T*_min_ = 0.332, *T*_max_ = 0.379
12347 measured reflections
4422 independent reflections
3995 reflections with *I* > 2σ(*I*)
*R*_int_ = 0.030

#### Refinement

*R*[*F* ^2^ > 2σ(*F* ^2^)] = 0.029
*wR*(*F* ^2^) = 0.054
*S* = 1.03
4422 reflections
291 parameters
H-atom parameters constrained
Δρ_max_ = 0.65 e Å^−3^
Δρ_min_ = −0.83 e Å^−3^

### 

Data collection: *CrystalClear* (Rigaku/MSC, 2006 ▶); cell refinement: *CrystalClear*; data reduction: *CrystalClear*; program(s) used to solve structure: *SHELXS97* (Sheldrick, 2008 ▶); program(s) used to refine structure: *SHELXL97* (Sheldrick, 2008 ▶); molecular graphics: *PLATON* (Spek, 2003 ▶); software used to prepare material for publication: *SHELXTL* (Sheldrick, 2008 ▶) and *PLATON*.

## Supplementary Material

Crystal structure: contains datablocks global, I. DOI: 10.1107/S1600536808040269/hk2534sup1.cif

Structure factors: contains datablocks I. DOI: 10.1107/S1600536808040269/hk2534Isup2.hkl

Additional supplementary materials:  crystallographic information; 3D view; checkCIF report

## Figures and Tables

**Table:** 

Hg1—N5^i^	2.295 (3)
Hg1—N1	2.337 (3)
Hg1—Cl1	2.3994 (12)
Hg1—Cl2	2.4249 (14)

**Table:** 

N5^i^—Hg1—N1	106.50 (11)
N5^i^—Hg1—Cl1	117.02 (8)
N1—Hg1—Cl1	108.03 (8)
N5^i^—Hg1—Cl2	103.20 (9)
N1—Hg1—Cl2	99.40 (8)
Cl1—Hg1—Cl2	120.60 (4)

Symmetry code: (i) 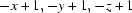.

**Table 2:** Hydrogen-bond geometry (Å, °)

*D*—H⋯*A*	*D*—H	H⋯*A*	*D*⋯*A*	*D*—H⋯*A*
N2—H2*A*⋯O3	0.86	2.32	3.090 (4)	149
N2—H2*A*⋯N3	0.86	2.27	2.692 (2)	110
N4—H4*A*⋯O3	0.86	2.06	2.870 (4)	156
N4—H4*A*⋯N3	0.86	2.33	2.714 (3)	107

**Fig. 1.**
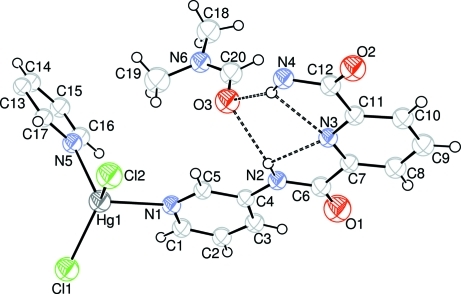
The asymmetric unit of the title molecule, with the atom-numbering scheme. Hydrogen bonds are shown as dashed lines.

## supplementary crystallographic information

### Comment

The expansion of the field of metal–organic frameworks (MOFs) of predetermined structure depends on the judicious choice of new linkers and nodes of appropriate coordination algorithms (Ockwig *et al.*,  2005). Rigid polydentate N-donor ligands are typical linkers employed in such a work. *N*,*N*'-bis(pyridin-3-yl)-2,6-pyridinedicarboxamide, with a rigid conjugated clamp-like configuration, is a convenient bridging ligand for the syntheses of cyclic complexes (Qin *et al.*,  2003; Baer *et al.*,  2002). In this work, we selected this ligand as linker, to generate the new title coordination complex, and we report herein its crystal structure.

The asymmetric unit of the title compound (Fig. 1) contains one-half molecule and an N,N-dimethylformamide (DMF) molecule, where the bond lengths (Allen *et al.*,  1987) and angles are within normal ranges. The Hg^II^ atom is four-coordinated by two N atoms from two ligands and two Cl atoms in a distorted tetrahedral coordination geometry (Table 1). The two Hg^II^ atoms are bridged with two *N*,*N*'-bis(pyridin-3-yl)-2,6-pyridinedicarboxamide ligands to form a porous MOF with 28-membered macroring. The pyridine rings A (N1/C1–C5), B (N3/C7–C11) and C (N5/C13–C17) are oriented at dihedral angles of A/B = 3.31 (3)°, A/C = 62.29 (3)° and B/C = 60.76 (3)°. The intramolecular N—H···O hydrogen bonds (Table 2, Fig. 1) may be effective in the stabilization of the structure.

In the crystal structure, the π–π contacts between the pyridine rings, Cg1—Cg2^i^ and Cg3—Cg3^ii^ [symmetry codes: (i) 2 - x, 1 - y, -z; (ii) -x, 2 - y, 1 - z, where Cg1, Cg2 and Cg3 are centroids of the rings A (N1/C1–C5), B (N3/C7–C11) and C (N5/C13–C17), respectively] may further stabilize the structure, with centroid–centroid distances of 3.629 (3) Å and 3.595 (3) Å.

### Experimental

For the preparation of the title compound, the ligand *N*,*N*'-bis-(pyridin-3-yl)-2,6-pyridinedicarboxamide (0.016 g, 0.05 mmol) in DMF (5 ml) was added dropwise to a solution of HgCl2 (0.028 g, 0.1 mmol) in methanol (5 ml). The precipitate was filtered and the resulting solution was allowed to stand at room temperature in the dark. After one week high quality colorless crystals were obtained and dried in air.

### Crystal data

**Table:**

[Hg_2_Cl_4_(C_17_H_13_N_5_O_2_)_2_]·2C_3_H_7_NO	*Z* = 1
*M**_r_* = 1327.82	*F*(000) = 640
Triclinic, *P*1	*D*_x_ = 1.951 Mg m^−^^3^
Hall symbol: -P 1	Mo *K*α radiation, λ = 0.71073 Å
*a* = 7.4947 (15) Å	Cell parameters from 3289 reflections
*b* = 12.262 (3) Å	θ = 2.9–26.0°
*c* = 13.284 (3) Å	µ = 7.08 mm^−^^1^
α = 79.79 (3)°	*T* = 294 K
β = 73.74 (3)°	Prism, colourless
γ = 76.21 (3)°	0.20 × 0.18 × 0.17 mm
*V* = 1130.2 (5) Å^3^	

### Data collection

**Table:**

Rigaku Saturn 724 diffractometer	4422 independent reflections
Radiation source: fine-focus sealed tube	3995 reflections with *I* > 2σ(*I*)
graphite	*R*_int_ = 0.030
Detector resolution: 28.5714 pixels mm^-1^	θ_max_ = 26.0°, θ_min_ = 2.9°
dtprofit.ref scans	*h* = −9→9
Absorption correction: numerical (*CrystalClear*; Rigaku/MSC, 2006)	*k* = −15→15
*T*_min_ = 0.332, *T*_max_ = 0.379	*l* = −16→16
12347 measured reflections	

### Refinement

**Table:**

Refinement on *F*^2^	Primary atom site location: structure-invariant direct methods
Least-squares matrix: full	Secondary atom site location: difference Fourier map
*R*[*F*^2^ > 2σ(*F*^2^)] = 0.029	Hydrogen site location: inferred from neighbouring sites
*wR*(*F*^2^) = 0.054	H-atom parameters constrained
*S* = 1.03	*w* = 1/[σ^2^(*F*_o_^2^) + (0.0195*P*)^2^ + 0.7349*P*] where *P* = (*F*_o_^2^ + 2*F*_c_^2^)/3
4422 reflections	(Δ/σ)_max_ = 0.002
291 parameters	Δρ_max_ = 0.65 e Å^−^^3^
0 restraints	Δρ_min_ = −0.83 e Å^−^^3^

### Special details

**Table:**

Geometry. All e.s.d.'s (except the e.s.d. in the dihedral angle between two l.s. planes) are estimated using the full covariance matrix. The cell e.s.d.'s are taken into account individually in the estimation of e.s.d.'s in distances, angles and torsion angles; correlations between e.s.d.'s in cell parameters are only used when they are defined by crystal symmetry. An approximate (isotropic) treatment of cell e.s.d.'s is used for estimating e.s.d.'s involving l.s. planes.
Refinement. Refinement of *F*^2^ against ALL reflections. The weighted *R*-factor *wR* and goodness of fit *S* are based on *F*^2^, conventional *R*-factors *R* are based on *F*, with *F* set to zero for negative *F*^2^. The threshold expression of *F*^2^ > σ(*F*^2^) is used only for calculating *R*-factors(gt) *etc*. and is not relevant to the choice of reflections for refinement. *R*-factors based on *F*^2^ are statistically about twice as large as those based on *F*, and *R*- factors based on ALL data will be even larger.

### Fractional atomic coordinates and isotropic or equivalent isotropic displacement parameters (Å^2^)

**Table:**

	*x*	*y*	*z*	*U*_iso_*/*U*_eq_	
Hg1	0.47943 (3)	0.144664 (13)	0.675659 (12)	0.04821 (7)	
Cl1	0.39934 (16)	−0.03890 (9)	0.72493 (9)	0.0566 (3)	
Cl2	0.26095 (18)	0.30392 (9)	0.61184 (9)	0.0611 (3)	
O1	0.2543 (5)	0.4774 (3)	1.1235 (2)	0.0738 (10)	
O2	−0.1219 (4)	0.9357 (2)	0.7985 (2)	0.0511 (7)	
O3	0.3641 (4)	0.5771 (2)	0.7182 (2)	0.0546 (8)	
N1	0.4906 (4)	0.2103 (2)	0.8281 (2)	0.0358 (7)	
N2	0.2824 (4)	0.4689 (2)	0.9507 (2)	0.0337 (7)	
H2A	0.2584	0.5087	0.8941	0.040*	
N3	0.1061 (4)	0.6871 (2)	0.9285 (2)	0.0316 (7)	
N4	0.0775 (4)	0.7801 (2)	0.7310 (2)	0.0352 (7)	
H4A	0.1452	0.7151	0.7459	0.042*	
N5	0.2361 (4)	0.8435 (2)	0.4443 (2)	0.0387 (7)	
N6	0.6833 (5)	0.5377 (3)	0.6475 (2)	0.0417 (8)	
C1	0.5503 (5)	0.1381 (3)	0.9066 (3)	0.0387 (9)	
H7	0.6118	0.0645	0.8944	0.046*	
C2	0.5222 (5)	0.1712 (3)	1.0037 (3)	0.0401 (9)	
H8	0.5634	0.1198	1.0569	0.048*	
C3	0.4326 (5)	0.2808 (3)	1.0236 (3)	0.0388 (9)	
H9	0.4124	0.3036	1.0897	0.047*	
C4	0.3738 (5)	0.3557 (3)	0.9418 (3)	0.0313 (8)	
C5	0.4047 (5)	0.3161 (3)	0.8456 (3)	0.0323 (8)	
H11	0.3639	0.3654	0.7911	0.039*	
C6	0.2281 (5)	0.5222 (3)	1.0382 (3)	0.0385 (9)	
C7	0.1299 (5)	0.6438 (3)	1.0247 (3)	0.0315 (8)	
C8	0.0694 (5)	0.7050 (3)	1.1103 (3)	0.0419 (9)	
H14	0.0886	0.6715	1.1759	0.050*	
C9	−0.0202 (5)	0.8169 (3)	1.0963 (3)	0.0432 (9)	
H15	−0.0601	0.8607	1.1519	0.052*	
C10	−0.0493 (5)	0.8622 (3)	0.9980 (3)	0.0384 (9)	
H16	−0.1121	0.9367	0.9867	0.046*	
C11	0.0159 (5)	0.7954 (3)	0.9168 (3)	0.0323 (8)	
C12	−0.0168 (5)	0.8443 (3)	0.8101 (3)	0.0346 (8)	
C13	0.0691 (5)	0.8157 (3)	0.6251 (3)	0.0324 (8)	
C14	−0.1022 (5)	0.8551 (3)	0.5975 (3)	0.0397 (9)	
H20	−0.2165	0.8604	0.6488	0.048*	
C15	−0.0995 (6)	0.8861 (3)	0.4925 (3)	0.0437 (10)	
H21	−0.2127	0.9115	0.4720	0.052*	
C16	0.0711 (6)	0.8796 (3)	0.4179 (3)	0.0424 (10)	
H22	0.0713	0.9010	0.3471	0.051*	
C17	0.2348 (5)	0.8112 (3)	0.5462 (3)	0.0341 (8)	
H23	0.3500	0.7847	0.5645	0.041*	
C18	0.8658 (6)	0.5671 (4)	0.6339 (4)	0.0574 (12)	
H24A	0.9104	0.5982	0.5620	0.086*	
H24B	0.9552	0.5005	0.6504	0.086*	
H24C	0.8526	0.6220	0.6802	0.086*	
C19	0.6793 (7)	0.4444 (4)	0.5953 (4)	0.0645 (13)	
H25A	0.5525	0.4308	0.6137	0.097*	
H25B	0.7630	0.3778	0.6174	0.097*	
H25C	0.7196	0.4629	0.5202	0.097*	
C20	0.5254 (6)	0.5959 (3)	0.7025 (3)	0.0470 (10)	
H26	0.5363	0.6565	0.7322	0.056*	

### Atomic displacement parameters (Å^2^)

**Table:**

	*U*^11^	*U*^22^	*U*^33^	*U*^12^	*U*^13^	*U*^23^
Hg1	0.06800 (12)	0.04056 (10)	0.03301 (9)	−0.01143 (8)	−0.00684 (7)	−0.00492 (7)
Cl1	0.0584 (7)	0.0423 (6)	0.0694 (7)	−0.0179 (5)	−0.0136 (6)	−0.0004 (5)
Cl2	0.0773 (8)	0.0504 (6)	0.0618 (7)	−0.0063 (6)	−0.0364 (6)	−0.0009 (5)
O1	0.125 (3)	0.0519 (19)	0.0363 (17)	0.0185 (19)	−0.0330 (18)	−0.0114 (14)
O2	0.0582 (18)	0.0368 (15)	0.0444 (16)	0.0123 (14)	−0.0092 (14)	−0.0039 (13)
O3	0.0463 (18)	0.0508 (18)	0.0482 (17)	0.0070 (14)	0.0008 (14)	−0.0013 (14)
N1	0.0415 (18)	0.0313 (16)	0.0329 (16)	−0.0063 (14)	−0.0069 (14)	−0.0047 (13)
N2	0.0470 (18)	0.0270 (15)	0.0231 (15)	0.0000 (13)	−0.0109 (13)	0.0006 (12)
N3	0.0299 (15)	0.0311 (16)	0.0310 (16)	−0.0052 (13)	−0.0035 (13)	−0.0043 (13)
N4	0.0411 (18)	0.0278 (15)	0.0287 (16)	0.0016 (13)	−0.0053 (13)	−0.0002 (13)
N5	0.050 (2)	0.0323 (16)	0.0319 (17)	−0.0067 (15)	−0.0090 (15)	−0.0024 (14)
N6	0.050 (2)	0.0349 (17)	0.0342 (17)	−0.0052 (15)	−0.0043 (15)	−0.0021 (14)
C1	0.039 (2)	0.0321 (19)	0.040 (2)	−0.0018 (16)	−0.0055 (17)	−0.0050 (17)
C2	0.044 (2)	0.036 (2)	0.037 (2)	−0.0031 (17)	−0.0159 (18)	0.0055 (17)
C3	0.050 (2)	0.036 (2)	0.031 (2)	−0.0041 (18)	−0.0150 (18)	−0.0041 (16)
C4	0.0338 (19)	0.0305 (18)	0.0276 (18)	−0.0051 (15)	−0.0075 (15)	−0.0006 (15)
C5	0.042 (2)	0.0294 (18)	0.0243 (18)	−0.0056 (16)	−0.0101 (15)	0.0007 (15)
C6	0.044 (2)	0.041 (2)	0.029 (2)	−0.0039 (18)	−0.0095 (17)	−0.0072 (17)
C7	0.0316 (19)	0.0341 (19)	0.0273 (18)	−0.0065 (15)	−0.0045 (15)	−0.0045 (15)
C8	0.045 (2)	0.051 (2)	0.029 (2)	−0.0106 (19)	−0.0050 (17)	−0.0096 (18)
C9	0.048 (2)	0.044 (2)	0.037 (2)	−0.0104 (19)	0.0008 (18)	−0.0206 (18)
C10	0.037 (2)	0.034 (2)	0.043 (2)	−0.0042 (16)	−0.0050 (17)	−0.0120 (17)
C11	0.0293 (18)	0.0325 (19)	0.0319 (19)	−0.0071 (15)	−0.0004 (15)	−0.0063 (16)
C12	0.033 (2)	0.032 (2)	0.035 (2)	−0.0030 (16)	−0.0058 (16)	−0.0025 (16)
C13	0.039 (2)	0.0212 (17)	0.035 (2)	−0.0014 (15)	−0.0110 (16)	−0.0010 (15)
C14	0.039 (2)	0.0318 (19)	0.048 (2)	−0.0065 (17)	−0.0102 (18)	−0.0053 (17)
C15	0.051 (2)	0.0291 (19)	0.059 (3)	−0.0043 (18)	−0.030 (2)	−0.0036 (19)
C16	0.064 (3)	0.029 (2)	0.040 (2)	−0.0092 (19)	−0.024 (2)	−0.0007 (17)
C17	0.039 (2)	0.0298 (18)	0.0311 (19)	−0.0017 (16)	−0.0099 (16)	−0.0016 (15)
C18	0.058 (3)	0.059 (3)	0.054 (3)	−0.019 (2)	−0.013 (2)	0.005 (2)
C19	0.058 (3)	0.053 (3)	0.074 (3)	−0.016 (2)	0.010 (2)	−0.024 (2)
C20	0.064 (3)	0.036 (2)	0.033 (2)	0.003 (2)	−0.012 (2)	−0.0016 (17)

### Geometric parameters (Å, °)

**Table:**

Hg1—N5^i^	2.295 (3)	C3—H9	0.9300
Hg1—N1	2.337 (3)	C4—C5	1.387 (5)
Hg1—Cl1	2.3994 (12)	C5—H11	0.9300
Hg1—Cl2	2.4249 (14)	C6—C7	1.501 (5)
O1—C6	1.215 (4)	C7—C8	1.383 (5)
O2—C12	1.219 (4)	C8—C9	1.381 (5)
O3—C20	1.238 (5)	C8—H14	0.9300
N1—C5	1.333 (4)	C9—C10	1.381 (5)
N1—C1	1.348 (5)	C9—H15	0.9300
N2—C6	1.349 (4)	C10—C11	1.378 (5)
N2—C4	1.404 (4)	C10—H16	0.9300
N2—H2A	0.8600	C11—C12	1.501 (5)
N3—C7	1.338 (4)	C13—C17	1.379 (5)
N3—C11	1.343 (4)	C13—C14	1.386 (5)
N4—C12	1.354 (4)	C14—C15	1.374 (5)
N4—C13	1.412 (4)	C14—H20	0.9300
N4—H4A	0.8600	C15—C16	1.377 (6)
N5—C16	1.332 (5)	C15—H21	0.9300
N5—C17	1.340 (4)	C16—H22	0.9300
N5—Hg1^i^	2.295 (3)	C17—H23	0.9300
N6—C20	1.320 (5)	C18—H24A	0.9600
N6—C19	1.449 (5)	C18—H24B	0.9600
N6—C18	1.451 (5)	C18—H24C	0.9600
C1—C2	1.365 (5)	C19—H25A	0.9600
C1—H7	0.9300	C19—H25B	0.9600
C2—C3	1.387 (5)	C19—H25C	0.9600
C2—H8	0.9300	C20—H26	0.9300
C3—C4	1.392 (5)		
			
N5^i^—Hg1—N1	106.50 (11)	C9—C8—H14	120.7
N5^i^—Hg1—Cl1	117.02 (8)	C7—C8—H14	120.7
N1—Hg1—Cl1	108.03 (8)	C10—C9—C8	118.6 (4)
N5^i^—Hg1—Cl2	103.20 (9)	C10—C9—H15	120.7
N1—Hg1—Cl2	99.40 (8)	C8—C9—H15	120.7
Cl1—Hg1—Cl2	120.60 (4)	C11—C10—C9	119.1 (4)
C5—N1—C1	119.2 (3)	C11—C10—H16	120.5
C5—N1—Hg1	118.8 (2)	C9—C10—H16	120.5
C1—N1—Hg1	120.8 (2)	N3—C11—C10	123.2 (3)
C6—N2—C4	127.2 (3)	N3—C11—C12	117.7 (3)
C6—N2—H2A	116.4	C10—C11—C12	119.1 (3)
C4—N2—H2A	116.4	O2—C12—N4	124.1 (3)
C7—N3—C11	117.0 (3)	O2—C12—C11	120.6 (3)
C12—N4—C13	122.6 (3)	N4—C12—C11	115.3 (3)
C12—N4—H4A	118.7	C17—C13—C14	118.5 (3)
C13—N4—H4A	118.7	C17—C13—N4	119.7 (3)
C16—N5—C17	118.8 (3)	C14—C13—N4	121.8 (3)
C16—N5—Hg1^i^	122.1 (2)	C15—C14—C13	118.6 (4)
C17—N5—Hg1^i^	118.7 (2)	C15—C14—H20	120.7
C20—N6—C19	120.9 (4)	C13—C14—H20	120.7
C20—N6—C18	121.5 (4)	C14—C15—C16	119.9 (4)
C19—N6—C18	117.6 (3)	C14—C15—H21	120.1
N1—C1—C2	121.1 (3)	C16—C15—H21	120.1
N1—C1—H7	119.4	N5—C16—C15	121.8 (4)
C2—C1—H7	119.4	N5—C16—H22	119.1
C1—C2—C3	120.6 (3)	C15—C16—H22	119.1
C1—C2—H8	119.7	N5—C17—C13	122.5 (3)
C3—C2—H8	119.7	N5—C17—H23	118.8
C2—C3—C4	118.1 (3)	C13—C17—H23	118.8
C2—C3—H9	120.9	N6—C18—H24A	109.5
C4—C3—H9	120.9	N6—C18—H24B	109.5
C5—C4—C3	118.3 (3)	H24A—C18—H24B	109.5
C5—C4—N2	117.5 (3)	N6—C18—H24C	109.5
C3—C4—N2	124.2 (3)	H24A—C18—H24C	109.5
N1—C5—C4	122.6 (3)	H24B—C18—H24C	109.5
N1—C5—H11	118.7	N6—C19—H25A	109.5
C4—C5—H11	118.7	N6—C19—H25B	109.5
O1—C6—N2	123.9 (4)	H25A—C19—H25B	109.5
O1—C6—C7	120.5 (3)	N6—C19—H25C	109.5
N2—C6—C7	115.5 (3)	H25A—C19—H25C	109.5
N3—C7—C8	123.5 (3)	H25B—C19—H25C	109.5
N3—C7—C6	117.1 (3)	O3—C20—N6	126.0 (4)
C8—C7—C6	119.5 (3)	O3—C20—H26	117.0
C9—C8—C7	118.7 (4)	N6—C20—H26	117.0
			
N5^i^—Hg1—N1—C5	99.7 (3)	C6—C7—C8—C9	179.8 (3)
Cl1—Hg1—N1—C5	−133.8 (2)	C7—C8—C9—C10	1.4 (6)
Cl2—Hg1—N1—C5	−7.2 (3)	C8—C9—C10—C11	−1.6 (6)
N5^i^—Hg1—N1—C1	−92.7 (3)	C7—N3—C11—C10	0.7 (5)
Cl1—Hg1—N1—C1	33.8 (3)	C7—N3—C11—C12	−178.3 (3)
Cl2—Hg1—N1—C1	160.5 (3)	C9—C10—C11—N3	0.6 (6)
C5—N1—C1—C2	0.9 (6)	C9—C10—C11—C12	179.6 (3)
Hg1—N1—C1—C2	−166.7 (3)	C13—N4—C12—O2	2.8 (6)
N1—C1—C2—C3	−0.7 (6)	C13—N4—C12—C11	−177.2 (3)
C1—C2—C3—C4	−0.4 (6)	N3—C11—C12—O2	168.8 (3)
C2—C3—C4—C5	1.2 (5)	C10—C11—C12—O2	−10.3 (5)
C2—C3—C4—N2	−179.7 (3)	N3—C11—C12—N4	−11.2 (5)
C6—N2—C4—C5	175.5 (4)	C10—C11—C12—N4	169.7 (3)
C6—N2—C4—C3	−3.6 (6)	C12—N4—C13—C17	129.3 (4)
C1—N1—C5—C4	0.0 (5)	C12—N4—C13—C14	−51.0 (5)
Hg1—N1—C5—C4	167.8 (3)	C17—C13—C14—C15	1.0 (5)
C3—C4—C5—N1	−1.0 (5)	N4—C13—C14—C15	−178.7 (3)
N2—C4—C5—N1	179.8 (3)	C13—C14—C15—C16	−1.2 (5)
C4—N2—C6—O1	0.8 (7)	C17—N5—C16—C15	1.1 (5)
C4—N2—C6—C7	−179.0 (3)	Hg1^i^—N5—C16—C15	−171.3 (3)
C11—N3—C7—C8	−0.9 (5)	C14—C15—C16—N5	0.1 (6)
C11—N3—C7—C6	179.1 (3)	C16—N5—C17—C13	−1.2 (5)
O1—C6—C7—N3	−179.9 (4)	Hg1^i^—N5—C17—C13	171.4 (3)
N2—C6—C7—N3	0.0 (5)	C14—C13—C17—N5	0.1 (5)
O1—C6—C7—C8	0.1 (6)	N4—C13—C17—N5	179.9 (3)
N2—C6—C7—C8	−180.0 (3)	C19—N6—C20—O3	2.2 (6)
N3—C7—C8—C9	−0.1 (6)	C18—N6—C20—O3	179.9 (4)

Symmetry codes: (i) −*x*+1, −*y*+1, −*z*+1.

### Hydrogen-bond geometry (Å, °)

**Table:**

*D*—H···*A*	*D*—H	H···*A*	*D*···*A*	*D*—H···*A*
N2—H2A···O3	0.86	2.32	3.090 (4)	149
N2—H2A···N3	0.86	2.27	2.692 (2)	110
N4—H4A···O3	0.86	2.06	2.870 (4)	156
N4—H4A···N3	0.86	2.33	2.714 (3)	107
